# Designing a Cruciform Specimen via Topology and Shape Optimisations under Equal Biaxial Tension Using Elastic Simulations

**DOI:** 10.3390/ma15145001

**Published:** 2022-07-18

**Authors:** Junxian Chen, Jianhai Zhang, Hongwei Zhao

**Affiliations:** 1 School of Mechanical and Aerospace Engineering, Jilin University, Changchun 130022, China; chenjunxianie5@gmail.com; 2 Key Laboratory of CNC Equipment Reliability, Ministry of Education, Jilin University, Changchun 130022, China; 3 Chongqing Research Institute, Jilin University, Chongqing 401120, China

**Keywords:** cruciform specimen, optimisation, topology, shape, finite element method, biaxial tensile test, material test

## Abstract

Stress uniformity within the gauge zone of a cruciform specimen significantly affects materials’ in-plane biaxial mechanical properties in material testing. The stress uniformity depends on the load transmission of the cruciform specimen from the fixtures to the gauge zone. Previous studies failed to alter the nature of the load transmission of the geometric features using parametric optimisations. To improve stress uniformity in the gauge zone, we optimised the cross-arms to design a centre-reduced cruciform specimen with topology and shape optimisations. The simulations show that the optimised specimen obtains significantly less stress variation and range in the gauge zone than the optimised specimen under different observed areas, directions, and load ratios of *von Mises, S*_11_, *S*_22_, and *S*_12_. In the quantified gauge zone, a more uniform stress distribution could be generated by optimizing specimen geometry, whose value should be estimated indirectly each time through simulations. We found that topology and shape optimisations could markedly improve stress uniformity in the gauge zone, and stress concentration at the cross-arms intersection. We first optimised the cruciform specimen structure by combining topology and shape optimisations, which provided a cost-effective way to improve stress uniformity in the gauge zone and reduce stress concentration at the cross-arms intersection, helping obtain reliable data to perform large strains in the in-plane biaxial tensile test.

## 1. Introduction

Cruciform specimens are widely used to evaluate materials’ in-plane biaxial mechanical properties, such as metals, ceramics, composites, and biomaterials. The structure of a cruciform specimen significantly affects stress uniformity in the gauge zone and the stress concentration at the intersection of the cross-arms. However, scholars failed to reach a consensus on the specimen’s structure [1]. Currently, only ISO 16842: 2021 regulates the structural form of metal cruciform specimens; the insufficient design standards of cruciform specimens lead to empirical designs of cruciform specimens, resulting in extensive data scatter in the in-plane biaxial tests. Therefore, we aimed to design an optimal structure of cruciform specimens to improve the reliability of the results in the in-plane biaxial testing.

The stress uniformity in the gauge zone of the cruciform specimen correlates with its structure [2,3,4,5,6,7,8,9]. The uneven stress distribution on the boundary of the gauge zone, and the stress concentration at the intersection of the cross-arms, deteriorate the stress uniformity in the gauge zone. The stress distribution of the boundary rests on the material distribution of the cross-arms. Therefore, different specimen forms have distinct stress distributions in the gauge zone, leading test data to vary from specimen to specimen. Improving the stress uniformity in the gauge zone reduces the test results’ dispersion. To obtain reliable test results, scholars devoted themselves to improving the stress uniformity in the gauge zone by optimising the structure of the cruciform specimen [10,11].

The finite element method (FEM) is a powerful tool for optimising cruciform specimens [12,13]. Studies prove that the structural design of the cruciform specimen structure using finite elements is cost-effective: the stress uniformity can be improved [8,14], and the boundary stress concentration can be reduced [15,16,17]. Before the experimental verification, most scholars determined the optimal design of the cruciform specimen via the finite element method combined with parameter optimisation methods [18,19,20]. Structural optimisation problems can be solved using either a parametric or non-parametric approach [21]. The parametric optimisation method can realise the optimal design of engineering structures by changing the parameterised structural elements, such as fillet and thickness, without changing the connectivity of the design domain. Although parameter optimisation can improve the stress uniformity and the stress concentration of the cruciform specimen, the sole change of parameters failed to alter the nature of the load transfer of geometric features [22,23].

The stress distribution in the gauge zone of the cruciform specimen rests on the nature of load transfer [11]. Many cruciform specimen structures were classified into three distinct types [24]: (i) inner concave fillets at the intersection of cross-arms, (ii) slots on the cross-arms, and (iii) reduced regions in the centre. All three specimens have stress concentrations at the cross-arm intersection, preventing large-strain plane biaxial testing [12]. Different test purposes should have different optimisation objectives, and different optimisation objectives produce different optimisation outcomes. The difficulties encountered in the design of cruciform specimens led us to identify two common problems that influence the design of cruciform specimens: the boundary form at the intersection of the cross-arms and the material distribution on the cross-arms. Only optimising the geometric features of cruciform specimens cannot provide the optimal design, since the parameter varies without changing the geometric features’ nature of load transfer [25,26]. To optimise the nature of load transfer, non-parametric optimisations should be used in the design of cruciform specimens. However, the optimal distribution of materials on the cruciform arms, and the optimal boundary shape at the intersection of the cross-arms, remain unclear.

To improve stress uniformity in the gauge zone, we redistributed the material on the four cruciform arms via topology optimisation, while optimising the cross-arms boundary via shape optimisation. Due to the two optimisations mentioned above, we designed a novel specimen with uniform stress distribution in the gauge zone of the specimen. Compared with the original specimen, the standard deviation of *von Mises* stress decreases from 6.91 MPa to 1.88 MPa when the ratio of the measurement area side length to gauge zone is 0.9; the stress range decreases from 20 MPa to 8 MPa at normalised distances of 0.1 or 0.9. We found that topology and shape optimisations improve stress uniformity in gauge zones, and reduce stress concentrations at intersections. The proposed method for optimising the cruciform specimen may help yield reliable biaxial test results in the plane by improving the uniformity of the stresses in the gauge zone, and reducing the probability of pre-failures caused by stress concentration at the intersection of the cross-arms.

## 2. Materials and Methods

Using finite element modelling (FEM), a centre-reduced cruciform specimen was constructed to improve the stress uniformity in the gauge zone for better characterising the nominal stress, and to reduce the stress concentration at the cross-arms to prevent failure first at the cross-arm intersection. Here outlines the original specimen, the optimisation process, topology optimisation, and shape optimisation.

### 2.1. Original Reduced Cruciform Specimen

**Figure 1:**
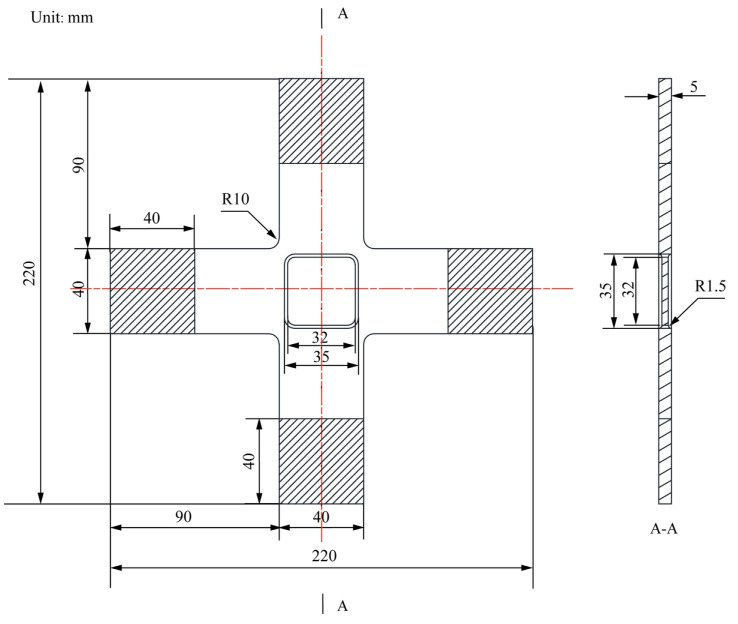
The geometry of the original cruciform specimen.

**Figure 2:**
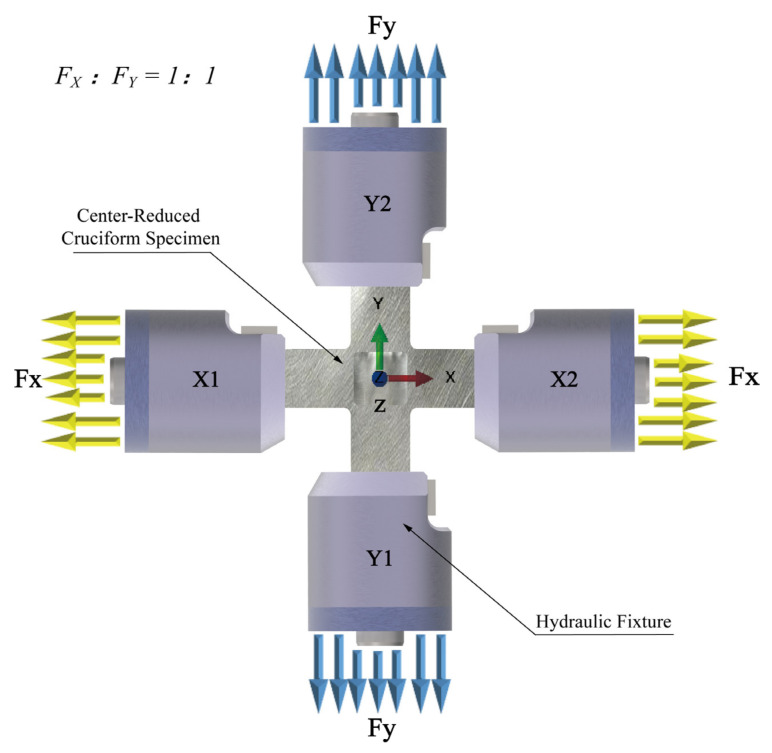
Schematic diagram of the equal biaxial tensile test.

The centre-reduced cruciform specimen (Figure 1) was constructed according to the in-plane biaxial testing system (Figure 2). Primary cruciform specimen dimensions considered the clamping part, specimen geometry, and centre-reduced area of the specimen. The clamping part has the dimensions of a clamping length of 40 mm, a clamping width of 40 mm, and a clamping thickness of 6 mm; the cruciform specimen has the following six primary dimensions: the overall length is 220 mm, the overall width is 220 mm, the rounded corners are 10 mm, the thickness is 6 mm, and the lengths of cross-arms are 90 mm. The reduced area has two primary dimensions: the centre-reduced zone is a square with a side length of 35 mm, and a reduced thickness of 1.5 mm on each side.

The specimen was optimised under equiaxial tensile conditions (*F*_x_/*F*_y_ = 1). Four actuators (X1, X2, Y1, and Y2) perpendicularly stretched the original cruciform specimen (Figure 2). The clamping ends of the cruciform specimens resisted the distributed shear forces generated by the hydraulic fixtures used during equiaxial tensile tests. Throughout the topology and shape optimisation process, a constant loading and clamping force was applied to the clamping end of the cruciform specimen. Our strategy was to optimise the topology and shape of the specimen sequentially, ensuring that the optimised specimen results from topology and shape optimisation. The geometric model for shape optimisation was derived from topology optimisation.

The specimen’s topology optimisation results in irregular boundary shapes, concentrating stress along the cross-arms. Due to the potential impact of stress concentration on the stress uniformity of the gauge zone, shape optimisation was used to improve the stress distribution on the boundary. As a result, we used shape optimisation following topology optimisation.

**Figure 3:**
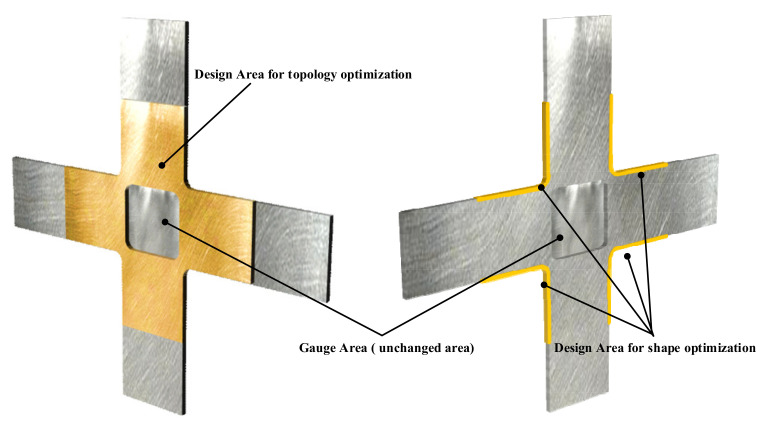
The design area optimised with topology optimisation and shape optimisation.

Figure 3 illustrates the application scenarios for topology and shape optimisations. Material distribution along the cross-arm was the object of the topology optimisation, in order to minimise strain energy in the design area. Four boundary areas were the object of shape optimisation, in order to reduce the maximum stress along the boundary area.

### 2.2. Optimisation Processes of Cruciform Specimen

**Figure 4:**
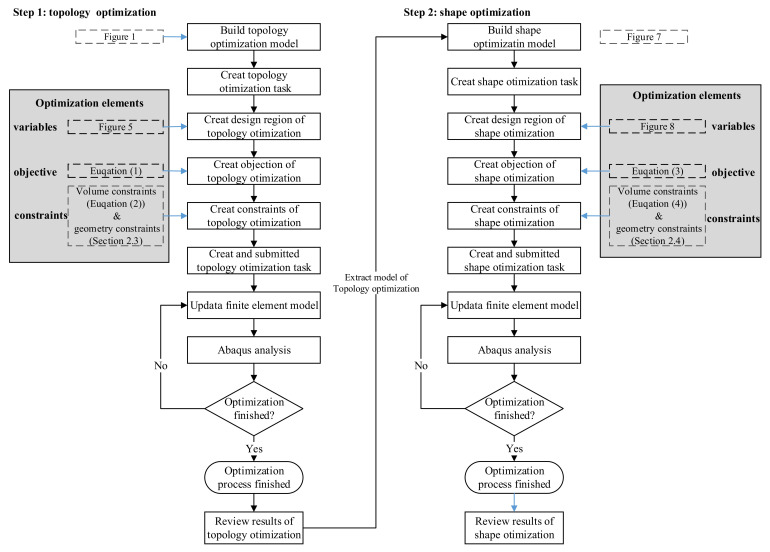
The optimised processes for a classic cruciform specimen.

Optimisations of the topology and shape were performed sequentially (Figure 4). Before performing topology optimisation, we established the cruciform specimen’s geometry, physical model, and finite element model sequentially, based on the primary geometric parameters of the classical cruciform specimen (Figure 1) and the requirements for the specimen’s in-plane biaxial tensile testing (Figure 2). We built the topology optimisation model based on the verified finite element model. We set the maximum number of optimisation iterations to 100 during the execution phase, performed topology optimisation, and extracted the topology-optimised model. Additionally, we developed original geometric, physical, and finite models for shape optimisation. The geometry model used in shape optimisation differed from the model used in topology optimisation. We set the maximum number of iterations to 100 during the execution phase of the optimisation process, performed shape optimisation, and extracted the shape-optimised model. Finally, we verified the stress uniformity of the optimised specimen, by using different areas and directions in the gauge zone, and the stress concentration at the intersection of the cross-arms.

### 2.3. Model of Topology Optimisation

Starting with the design area, which represents the maximum allowed area for the component, and with the boundary conditions, such as loads, fixtures, and manufacturing conditions, the optimisation system determines a new material distribution by removing and adding material in the design area [27].

**Figure 5:**
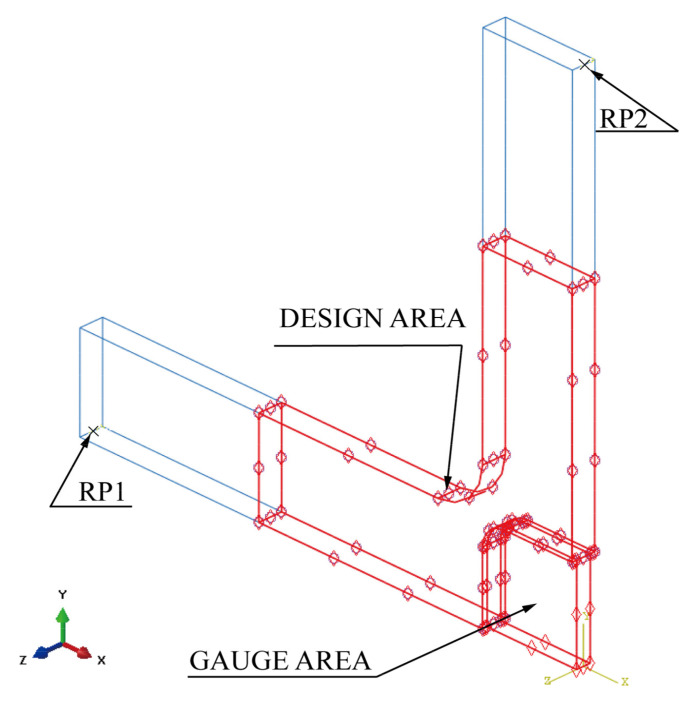
Quarter physical model for topology optimisation.

Topology optimisation could enhance stress uniformity in the gauge zone by redistributing the material on the cross arms. Figure 5 shows a physical topology optimisation model comprising the clamping ends, the design area, and the gauge zone. The clamping ends withstand loads of hydraulic fixtures.

**Figure 6:**
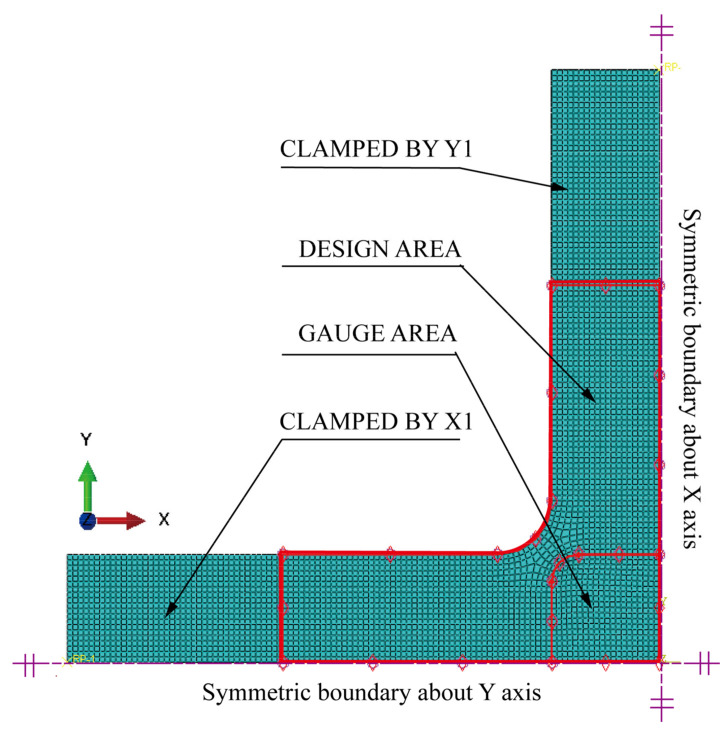
The finite element model for topology optimisation.

A quarter model of the classic structure was adopted, based on the symmetries of the geometry and force boundary conditions about the X and Y axes (Figure 5). The finite element model consists of the following (Figure 6):

1. Parts: the model consists of a single part built according to Figure 1 and Figure 5;
2. Mesh: the type of hexahedral element is C3D8R, and the total number of nodes and elements is 26,456 and 20,925, respectively (Figure 6). The five-element layers were arranged along the *Z*-axis (the thickness direction);
3. Materials: the Young’s modulus, yield strength, and Poisson’s ratio of HC1200 are 210 GPa, 1200 MPa, and 0.3, respectively;
4. Steps: only one step is specified. Nonlinear geometric effects are considered;
5. Loads: we rigidly coupled the clamping parts X1 and X2 with two reference points, RP1 and RP2 (Figure 5), respectively, to apply distributed force on the clamping parts with the concentrated forces on RP1 and RP2. One load of magnitude 40 kN is specified in RP1 and RP2;
6. Boundary conditions: symmetry boundary conditions are applied to specified regions (Figure 6). In addition, only the degrees of freedom U1 of RP1 and U2 of RP2 are released, and all the other degrees of freedom are fixed.

Configuring a topology optimisation analysis using Abaqus (Figure 4):

1. Creating an optimisation task: switch to the optimisation module. In the create optimization task dialogue box: Set the type of optimisation to topology and select the set DESIGN area, as shown in Figure 6;
2. In the basic tabbed page, select freeze boundary condition regions: the nodes clamped by the X1 fixture and X2 fixture; the nodes on the symmetry edges to the symmetrical planes (Figure 6);
3. Select specify smoothing region, and select the whole model;
4. Select fix all as the number of node layers adjoining the task region to remain free;
5. In the mesh smoothing quality tabbed page, set the target mesh quality to medium;
6. Creating design responses: design responses are parameters that are used to define the objective function or the constraints. In the create design response dialogue box: accept single-term as the type, and select whole model as the design response region;
7. In the edit design response dialogue box, select stress and Mises hypothesis (the field operator on values across steps and load cases is set to maximum value by default);
8. Creating an objective function: in the edit objective function dialogue box, add all design responses eligible to participate in an objective function, and change the target to minimise the maximum design response values (Equation (1));
9. Creating a constraint: select volume for the design response, toggle on A fraction of the initial value, and enter 0.9 (Equation (2));
10. Create the stamping geometric restriction: creating a demold control geometric restriction with the stamping technique to ensure that the final design proposal can be manufactured by removing material completely in a specified direction; we define a demold control geometric restriction using the stamping technique:
  - In the create geometric restriction dialogue box, select the whole model as the demold control region;
  - Change the demold technique to stamping and define the pull direction via the vector (0,0,1), which defines the global *Z*-axis as the pull direction.
11. Submit an optimisation process for the optimisation task optimise-shape. Note that the maximum cycles field is set to 100 by default for topology optimisation.

Topology optimisation is a procedure that yields an optimal material layout given a constraint while minimising/maximising an objective. Specifically, optimisation produces the stiffest possible material layout given a volume constraint. Thus, we set up a topology optimisation task that minimises the sum of strain energies over all elements in the cruciform specimen (equivalent to maximising the stiffness) (Equation (1)), given a relative volume constraint of 0.9 (Equation (2)).

The following is the objective of topology optimisation:

(1) $$\min\left\{\sum E(\rho_{e},u_{e})\right\},$$

where E is the strain energy of each element in the design area; $\rho_{e}$ is the reduction factor of the material density of each element in the design area; and $u_{e}$ is the displacement of each element in the design area.

The following are the constraints of topology optimisation:

(2) $$\sum_{e=1}^{n}\nu_{e}\rho_{e}\leq \alpha V_{initial},\;0\leq \rho_{e}\leq 1,$$

where $\nu_{e}$ is the volume of each element in the design area; α is the friction coefficient of the design area’s initial volume; and $V_{initial}$ is the displacement of each element in the design area.

### 2.4. Model of Shape Optimisation

Shape optimisation allows automatisation of this improvement process. The surface geometry of a given FE model is modified iteratively based on the FE results, such that the required optimisation target is reached. The start model is taken from an existing design, which should be improved, or from a previous topology optimisation [28,29].

**Figure 7:**
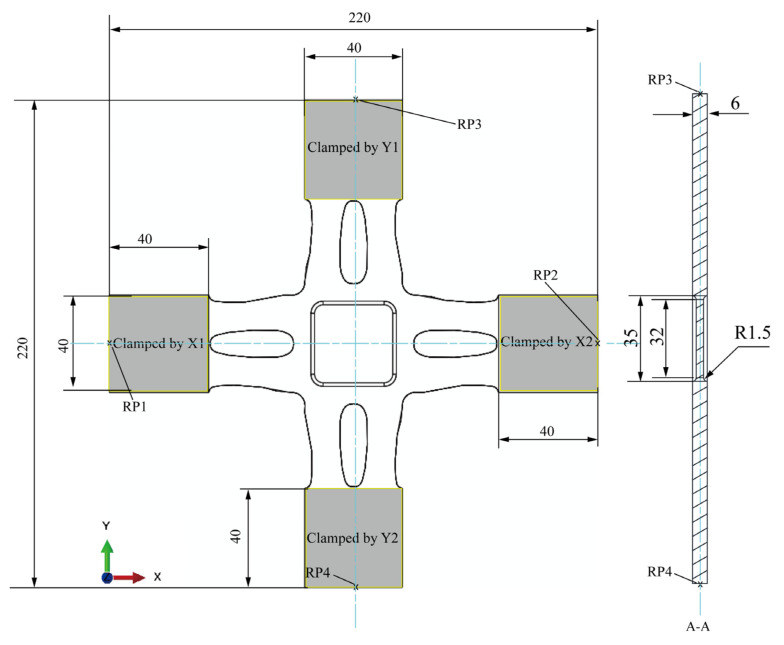
A physical model for shape optimisation.

The position of the nodes in the design area can be fine-tuned using shape optimisation to improve stress uniformity in the gauge area. The clamping ends, the cross arms, the boundaries, and the gauge zone comprise the physical model of shape optimisation (Figure 7). The optimal results from the topology optimisation determine the geometry of the cross arms (Figure 7). The following are the three components of shape optimisation: the objective is to minimise the maximum *von Mises* stress within the optimisation region, the variable is the shape of the boundary, and the constraint is to maintain the volume of the optimisation region.

**Figure 8:**
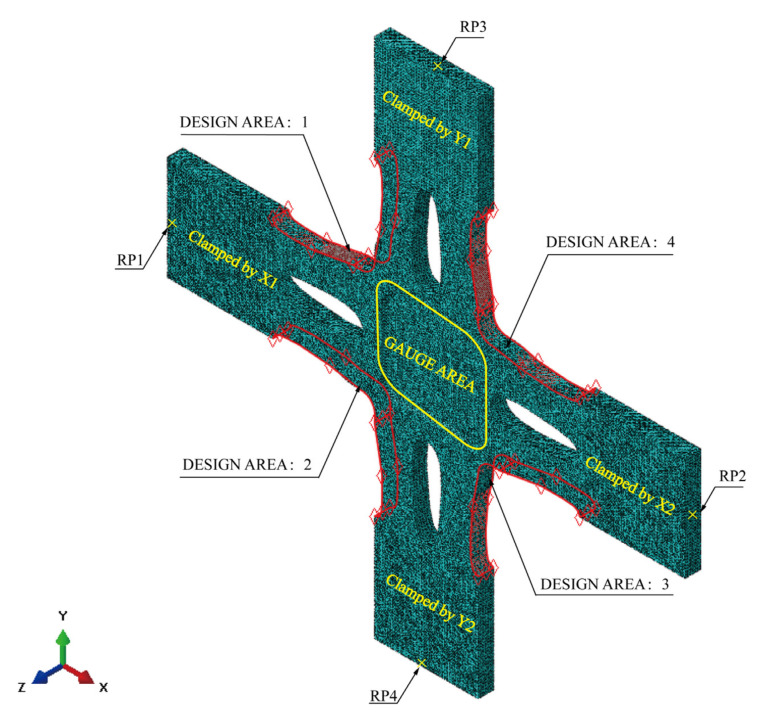
The finite element model for shape optimisation.

We developed the shape optimisation model based on the above finite element model (Figure 8). In Abaqus’ shape optimisation module, we specified an optimisation region, an objective, and a constraint to optimise. The design region consists of the nodes at the boundary of the optimisation region; the optimisation objective is to reduce the maximum Mises stress at the nodes of the optimisation region, and the optimisation constraint is the constant volume of the cross-arm. To prevent mesh distortion, the mesh co-ordinately deforms with the boundary. Other settings include: freezing the elements of clamping ends; and orienting the *Z*-axis in the direction of element removal.

The finite element model consists of the following (Figure 7):

1. Parts: the model consists of a single part, built according to Figure 4 and Figure 5;
2. Mesh: the type of hexahedral element is C3D10, and the total number of nodes and elements is 532,733 and 778,503, respectively (Figure 6). The five-element layers are arranged along the *Z*-axis (the thickness direction);
3. Materials: the Young’s modulus, yield strength, and Poisson’s ratio of HC1200 are 210 GPa, 1200 MPa, and 0.3, respectively;
4. Steps: only one step is specified. Nonlinear geometric effects are considered;
5. Loads: we rigidly coupled the clamping parts X1, X2, X3, and X4 with four reference points, RP1, RP2, RP3, and RP4 (Figure 8), respectively, to apply distributed force on the clamping parts with the concentrated forces on four reference points. One load of magnitude 40 kN is specified;
6. Boundary conditions: only the degrees of freedom U1 of RP1 and RP2, and U2 of RP3 and RP4, are released, and the other degrees of freedom are fixed.

Configuring a shape optimisation analysis using Abaqus (Figure 4):

1. Creating a shape optimisation task: switch to the optimisation module. In the create optimization task dialogue box, set the type of optimisation to shape and select the set DESIGN AREA: 1–4, as shown in Figure 8;
2. Select freeze boundary condition regions in the basic tabbed page: the nodes clamped by the X1, X2, Y1, and Y2 fixtures (Figure 8);
3. Select specify smoothing region, and select the whole model;
4. Select fix all as the number of node layers adjoining the task region to remain free;
5. In the mesh smoothing quality tabbed page, set the target mesh quality to medium;
6. Creating design responses: in the create design response dialogue box, accept single-term as the type, and select whole model as the design response region;
7. In the edit design response dialogue box, select stress and Mises hypothesis (the field operator on values across steps and load cases is set to maximum value by default);
8. Creating an objective function: in the edit objective function dialogue box, add all design responses eligible to participate in an objective function and change the target to minimise the maximum design response values (Equation (3));
9. Creating a constraint: select volume for the design response, toggle on A fraction of the initial value and enter 1 (Equation (4));
10. Create the stamping geometric restriction:
  - Choose the entire model as the demold control region in the dialogue box for creating geometric restrictions;
  - Assign the demold technique to stamping, and define the pull direction via the vector (0,0,1), which identifies the global Z-axis as the pull axis.
11. Submit an optimisation process for the optimisation task optimise-shape. Note that the maximum cycle field is set to 100 for shape optimisation.

Typically, in shape optimisation, the goal is to homogenise the stress on the surface of a component by adjusting the surface nodes (moving them inward or outward). Thus, minimisation is achieved by homogenisation. Shape optimisation is not limited to minimising stresses; it may be extended to plastic strains, natural frequencies, etc. This research will homogenise the Mises stress on the cross-arms intersection. The following is the objective of the shape optimisation:

(3) $$Min:\sigma_{von\_mises},$$

where *σ_von_mises_* is the *von Mises* stress of each node in the DESIGN AREA (Figure 8).

The purpose of creating volume constraints in shape optimisation is to ensure that the overall volume of the component remains the same. In some cases, adding material to reduce stress might be undesirable. In such cases, we can redistribute the material to minimise the stress. Volume constraints ensure that either no material is added, or very little material is added, due to the shape optimisation. The following is the volume constraints of the shape optimisation:

(4) $$V_{\mathrm{final}}=V_{\mathrm{initial}},$$

where ***V*_initial_** is the initial volume of all elements, and ***V*_final_** is the final volume of all elements.

## 3. Results

Original, topology-optimised, and topology-optimised specimens combined with shape-optimised specimens were designated type A, type B, and type C, respectively. The three specimens subjected to equal biaxial tensile stress exhibit significantly different stress distributions in the gauge zone. Compared with the stress distributions in the specimens’ areas, directions, and loads above, we demonstrate that the optimised specimen effectively improves the stress uniformity in the gauge zone and stress concentrations at cross-arm intersections.

### 3.1. Von Mises Stress Distribution of the Specimen

**Figure 9:**
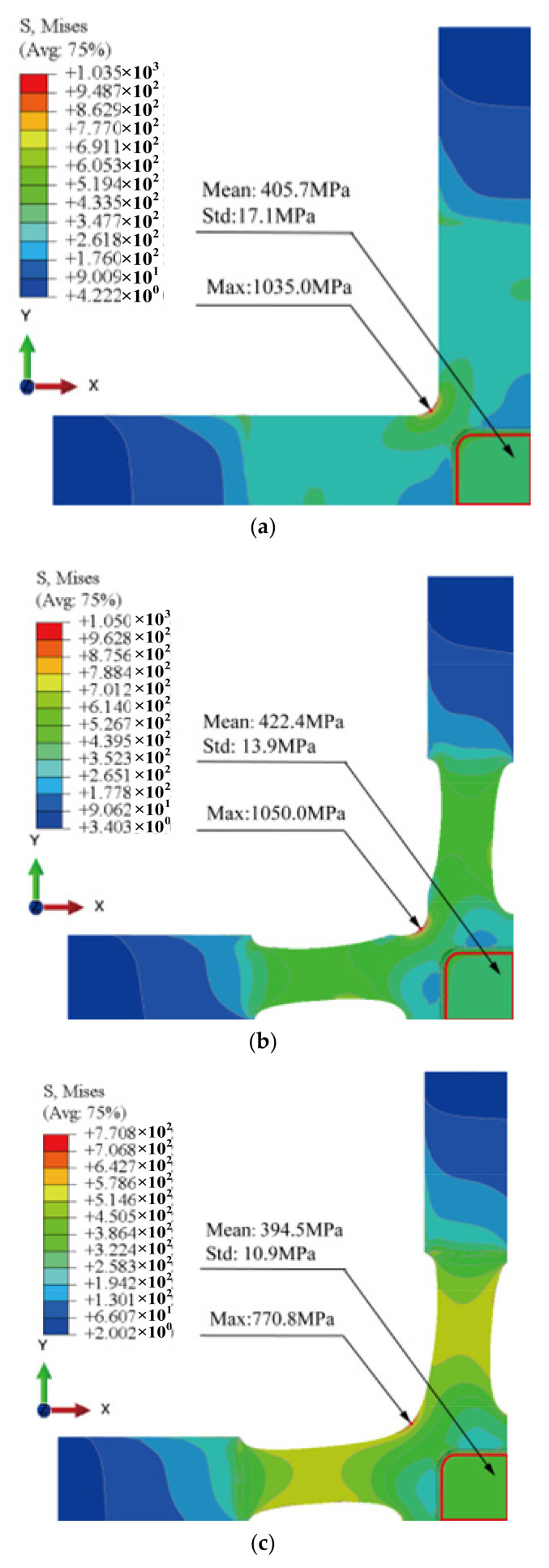
Stress distribution of the three specimens. (**a**) Type A: optimised specimen; (**b**) type B: specimen with only topology optimization; (**c**) type C: specimen with topology combined with shape optimisations.

Compared with type A (Figure 9a), the material of type B is redistributed in the cross-arms and boundaries (Figure 9b). Type C further reduces the stress concentrations at the intersection of the cross-arms (Figure 9c).

Although type B maintains the stress concentration at the cross-arms intersection (1050.0 MPa) in a similar way to type A (1035.0 MPa), the stress distribution in the gauge zone is redistributed due to the material redistribution. Additionally, we performed shape optimisations based on type B, such that the maximum *von Mises* stress of type C at the cross-arms intersection decreases from 1050.0 MPa to 770.8 MPa.

Type C exhibits *von Mises* stress’s lowest standard deviation (10.9 MPa) in the entire gauge zone, followed by type B (13.9 MPa). Compared with the above two optimised specimens (type B and type C), type A exhibits a significantly higher standard deviation (17.1 MPa).

### 3.2. Stress in Gauge Zone

**Figure 10:**
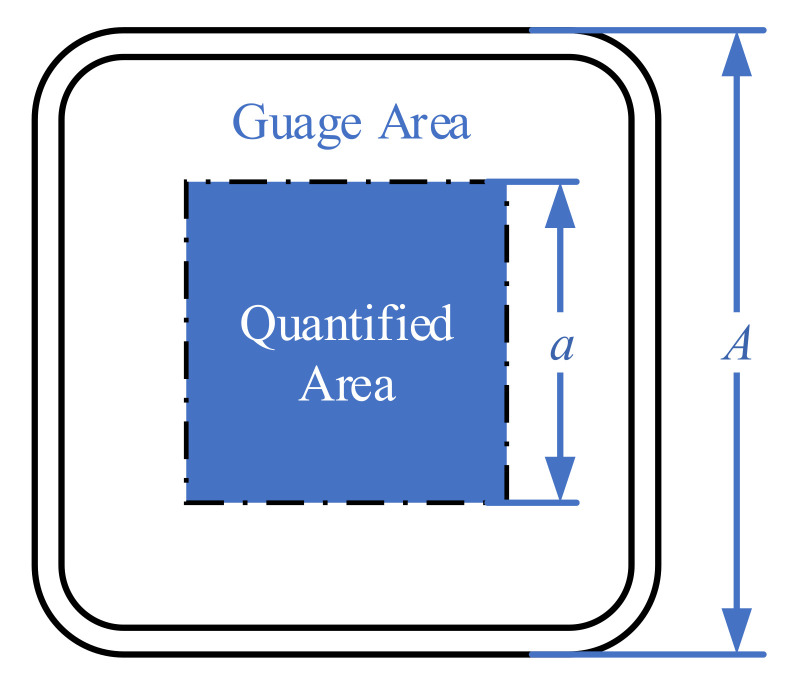
Quantified area of average and standard variation of stresses in the gauge zone.

To assess the stress uniformity in the gauge zone, we calculated the average and standard deviation of the stresses (*von Mises*, *S*_11_, *S*_22_, and *S*_12_) within a square area centred on the origin (Figure 10). A length ratio of 0.9 (a/A) was used as the quantified area to mitigate the effects of stress concentration along the gauge zone boundary.

**Figure 11:**
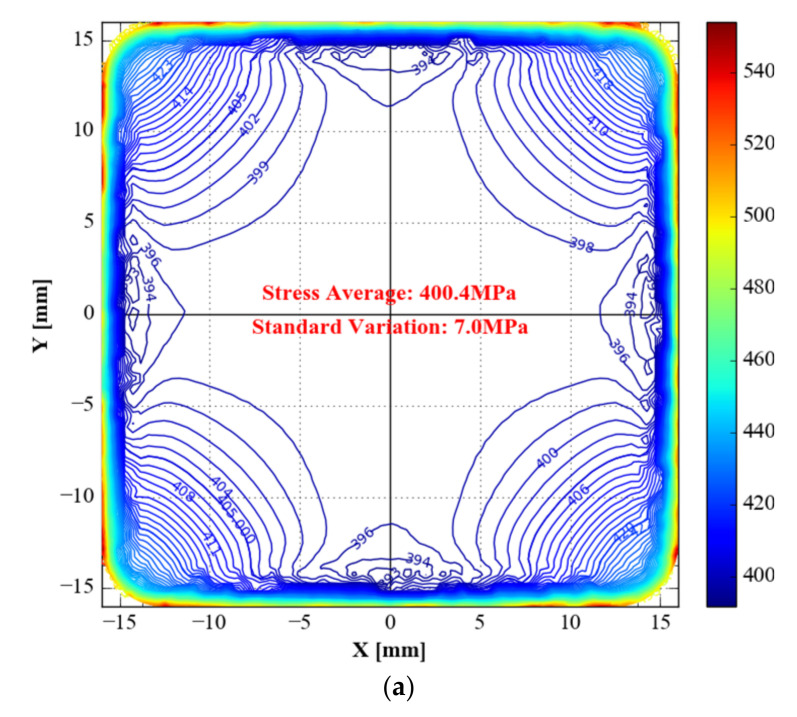

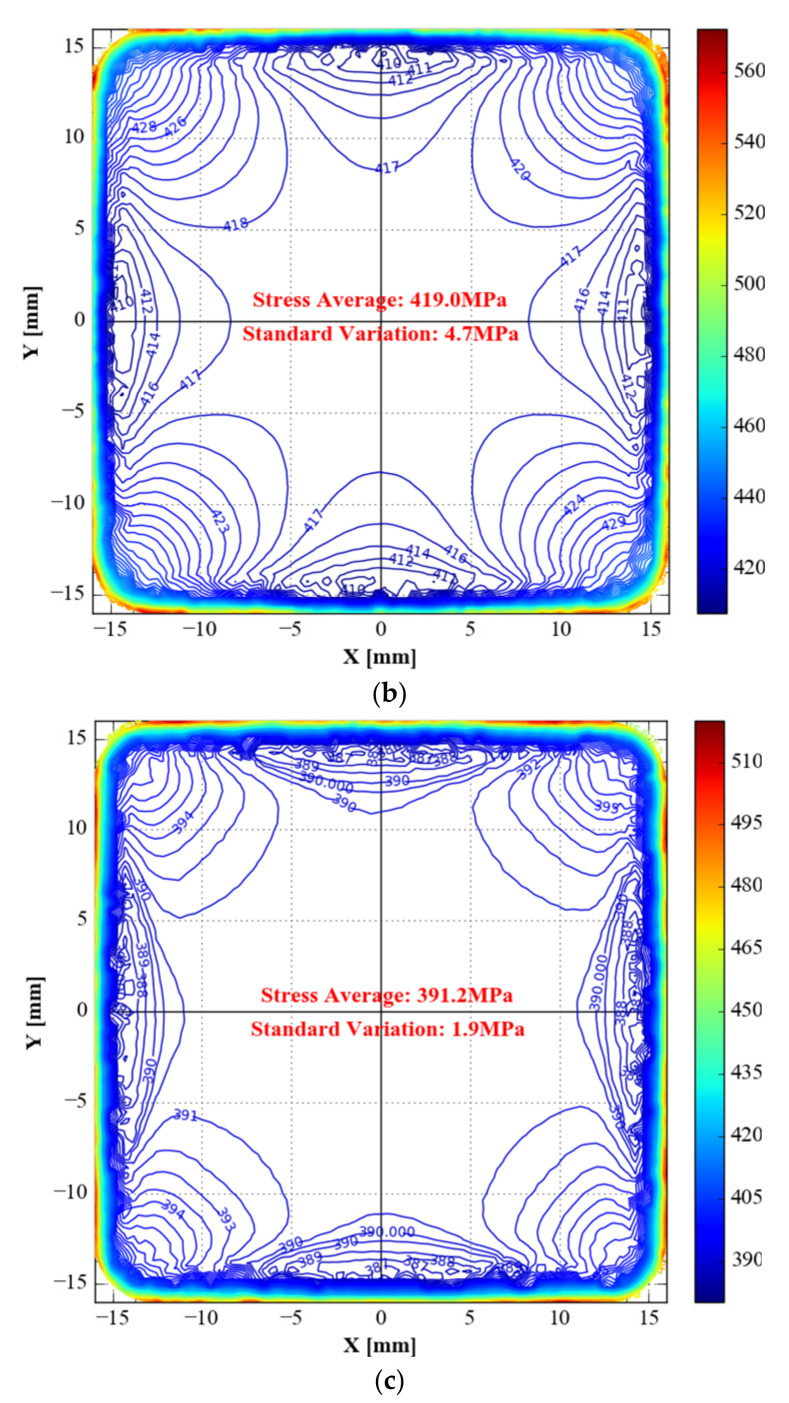
*von Mises* stress of the three specimens. (**a**) Type A: optimised specimen; (**b**) type B: specimen with topology-only optimization; (**c**) type C: specimen with topology combined with shape optimisations.

The contour diagram of *von Mises* has 1 MPa spacing between consecutive contour lines (Figure 11). Due to the slightest standard variation in the gauge zone, type C has the best stress homogeneity for *von Mises* stress, followed by type B. Type A specimens demonstrate significantly higher stress standard variation than the optimised specimens (type B and type C).

**Figure 12:**
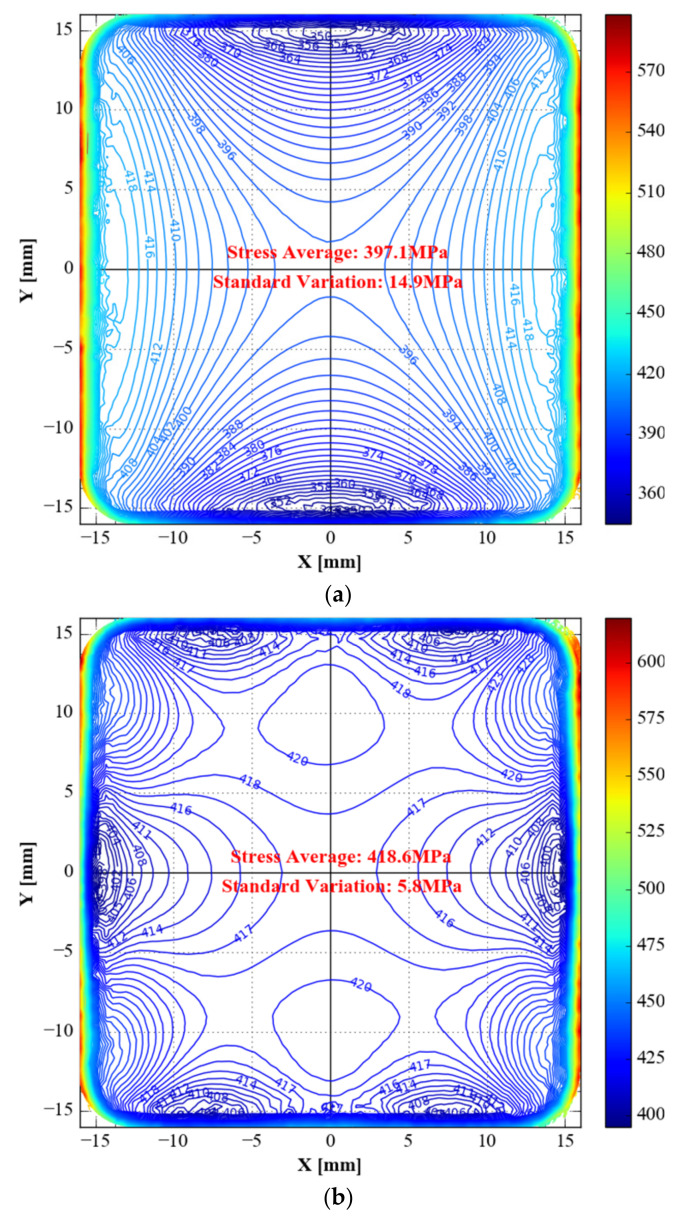

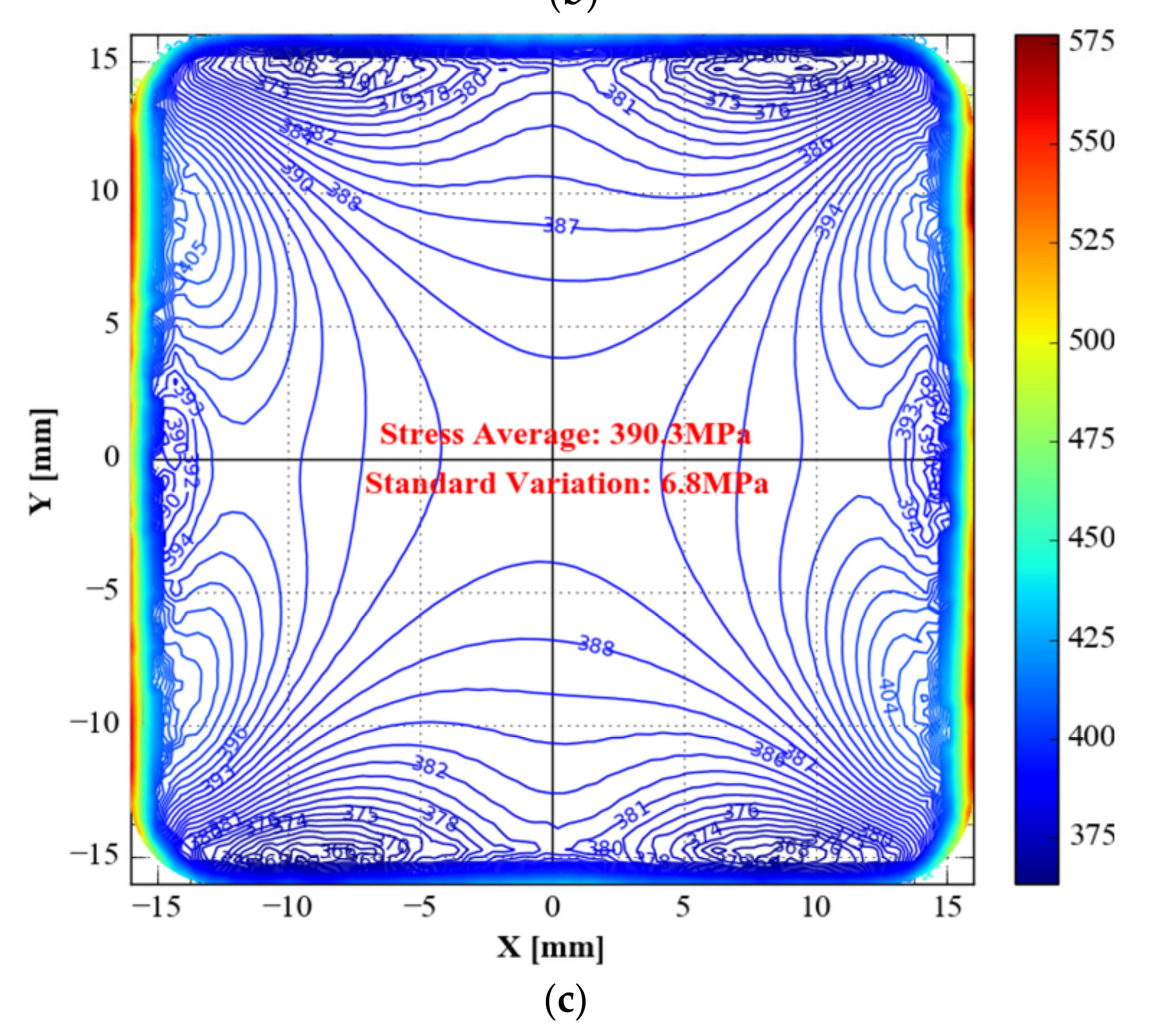
*S*_11_ of the three specimens in the gauge zone. (**a**) Type A: unoptimised specimen; (**b**) type B: specimen with topology-only optimization; (**c**) type C: specimen with topology combined with shape optimisations.

As the straight lines of *S*_22_ and *S*_11_ are symmetrical in the 45° direction across the origin, we list only the isoline of *S*_11_. Since the stress range of the original specimen *S*_11_ is too large compared to the optimised specimen, drawing with the same isoline interval results in an isoline interval that is too near. Thus, the distance between the two contours in the original *S*_11_ isoline map is 2 MPa, whereas the distance between the two isolines in the *S*_11_ isoline of type B and type C is 1 MPa (Figure 12).

The original specimen’s distribution of *S*_11_ is in the shape of a waist drum. The optimised specimen’s distribution is in the shape of a strip. The optimised specimen’s distribution is a combination of waist drum and strip. Even though the interval of the original specimen’s isoline is twice that of the two isolines in the *S*_11_ isoline of topology optimisation and topology optimisation combined with shape optimisation, the stress gradient of the original specimen’s isoline is also significantly greater than the stress gradient of the isoline of topology optimisation and topology optimisation combined with shape optimisation.

The topology-optimised specimen has the lowest standard deviation (5.8 MPa) in the observation area, followed by the sample optimised by topology combined with shape (6.8 MPa). The original specimen’s standard deviation (14.9 MPa) is greater than the two optimised specimens mentioned above.

Since the original specimen in S_12_ has an excessive variation range, drawing the same isoline interval results in a dense isoline interval. Thus, the distance between two contours in the original *S*_11_ isoline map is 8 MPa, whereas the distance between two isolines in the *S*_11_ isoline of topology optimisation and topology optimisation combined with shape optimisation is 5 MPa.

**Figure 13:**
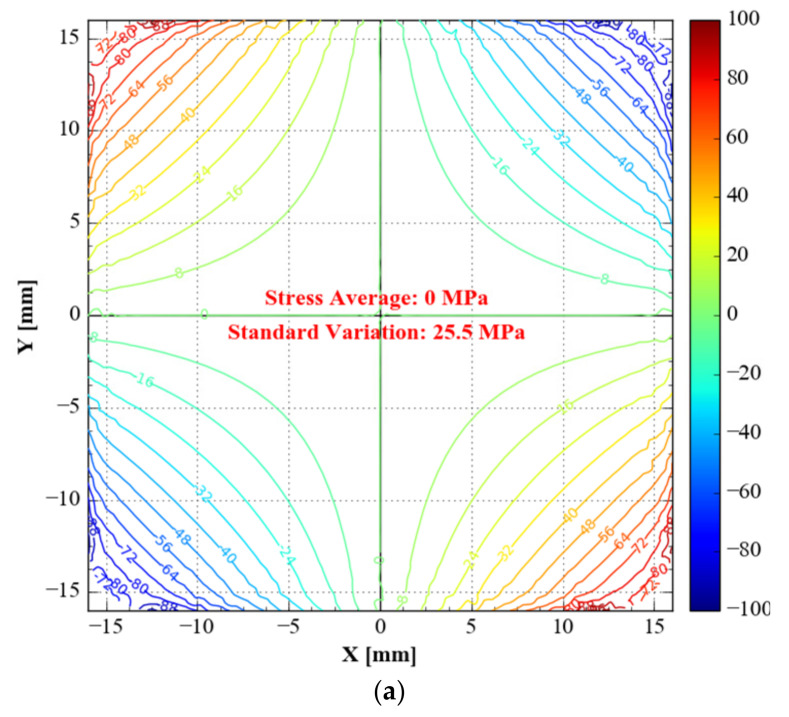

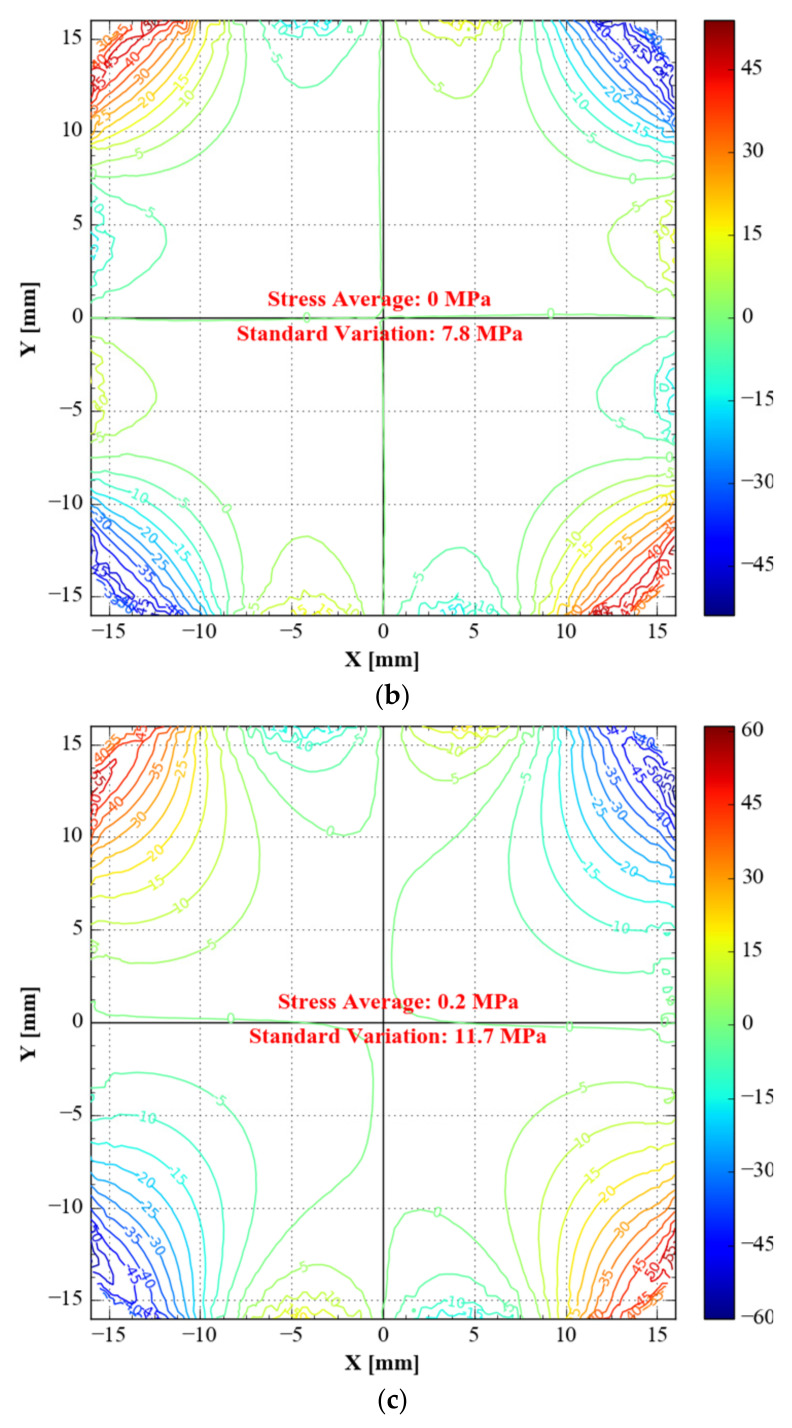
*S*_12_ of the three specimens in the gauge zone. (**a**) Type A: unoptimised specimen; (**b**) type B: specimen with topology-only optimization; (**c**) type C: specimen with topology combined with shape optimisations.

Type B has the slightest standard deviation (7.8 MPa), followed by type C (11.7 MPa). Type A (25.5 MPa) has a more significant standard deviation than the optimised specimens above (Figure 13).

The results show that the standard deviation of the stress of the three specimens decreases in turn in the same area (Table 1). Hence, the stress uniformity in the distance zone of the specimens optimised by topological shape gradually increases. Notably, the average stress values of the three specimens are different. The average stress after topological optimisation is higher than that of the original specimens, and the original specimens of the arm after shape optimisation are lower. The change in geometric parameters causes this change. Different structural loads of the cruciform specimens have different efficiencies in transferring from the clamp to the distance.

### 3.3. Stress along Directions

**Figure 14:**
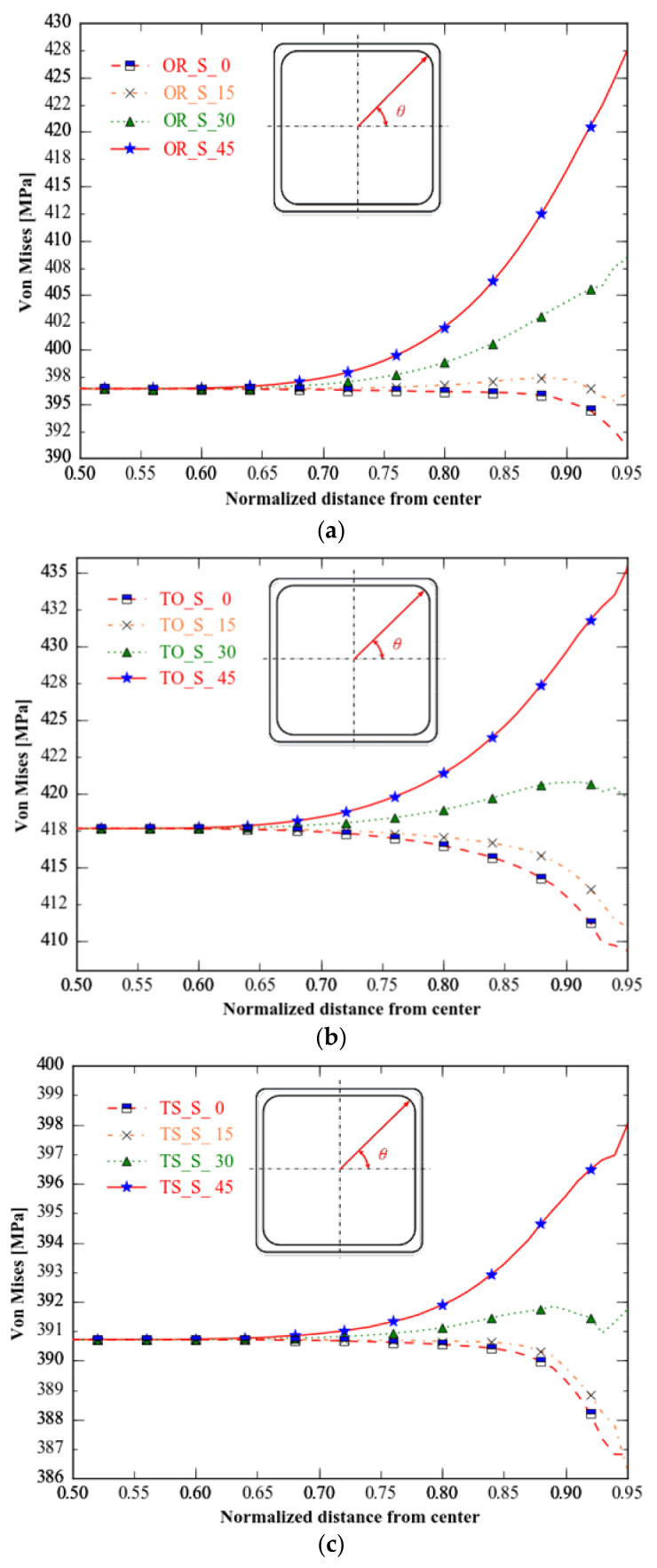
*von Mises* of the three specimens in four directions. (**a**) Type A: unoptimised specimen; (**b**) type B: specimen with topology-only optimization; (**c**) type C: specimen with topology combined with shape optimisations.

The *von Mises*, *S*_11_, *S*_22_, and *S*_12_ values were retrieved to examine how the gauge zone’s stress distribution varies with direction (Figure 14). We choose the *von Mises*, *S*_11_, and *S*_22_ distributions along a line segment whose normalised distance varies from 0.5 to 0.95 as the observation object, because of the distribution symmetry and the stress concentration near the gauge zone boundary.

The *von Mises* distributions at 0–45° and 45–90° about the 45° axis through the origin are symmetric, so we calculate only the *von Mises* stress in the 0–45° direction.

While all three specimens have a comparable *von Mises* distribution from 0 to 45 degrees, their stress ranges vary considerably. Type C has the narrowest stress range, followed by type B; type A has a more comprehensive stress range than the optimum specimens.

**Figure 15:**
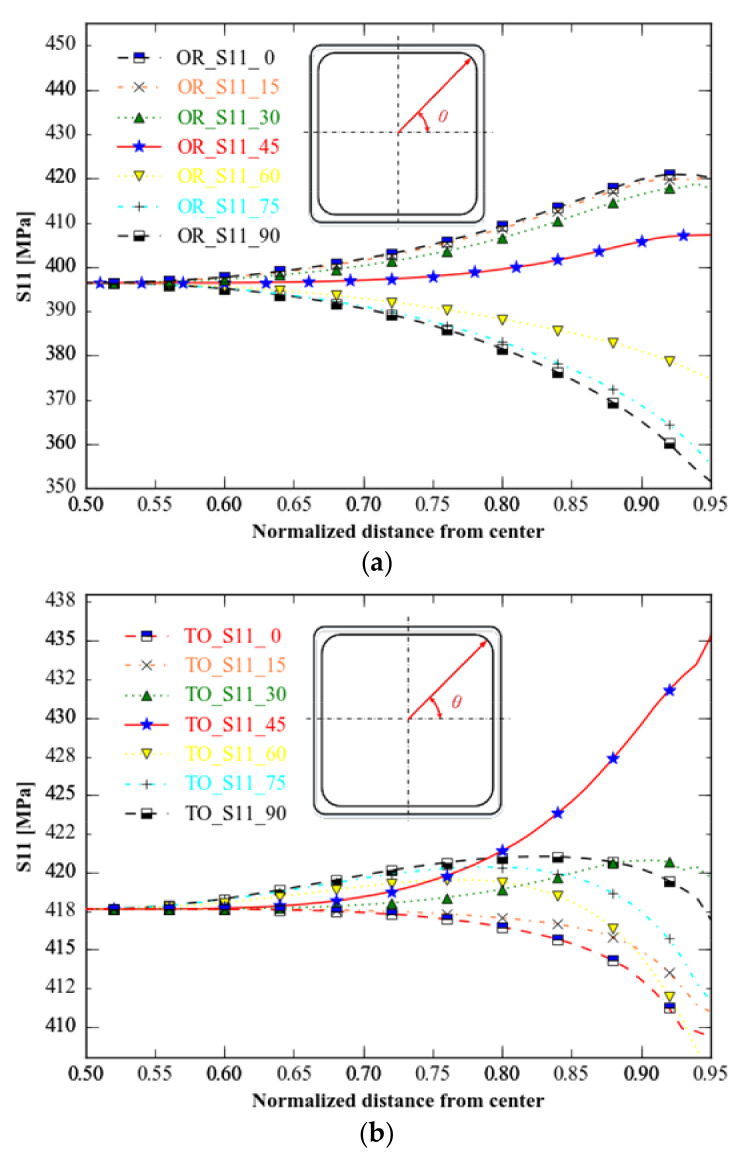

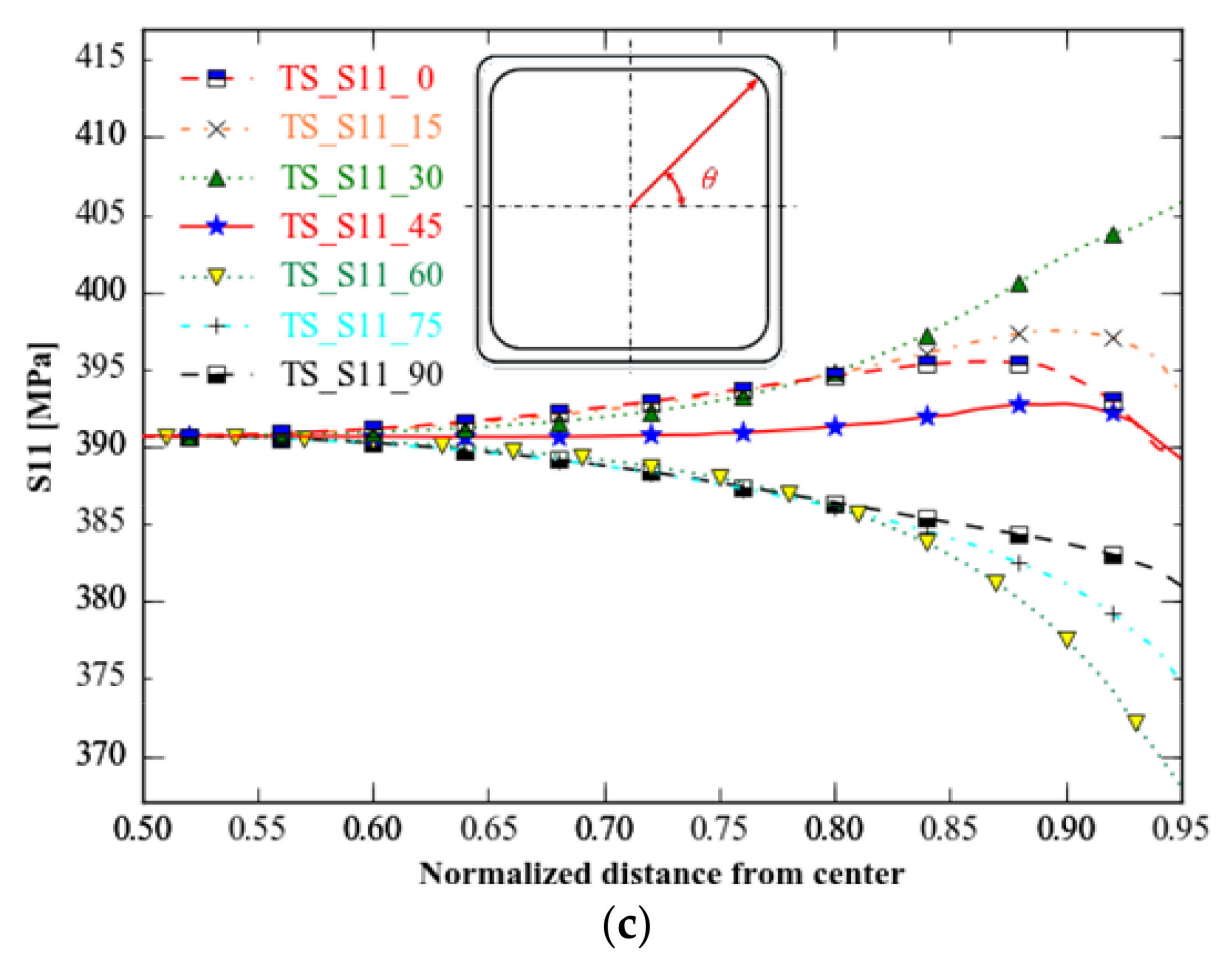
*S*_11_ of the three specimens in nine directions. (**a**) Type A: unoptimised specimen; (**b**) type B: specimen with topology-only optimization; (**c**) type C: specimen with topology combined with shape optimisations.

As the straight lines of *S*_11_ and *S*_22_ are symmetrical in the 45 degree direction across the origin under equiaxial tension, we sketch the stress distribution of *S*_11_ along 0°and 90° (Figure 15).

Compared with the distribution of *von Mises* in different directions, the distribution of *S*_11_ in the marking distance area of three different specimens is different in different directions: the original specimen’s maximum stress change direction is 0 and 90 degrees, topology optimisation is 45 degrees, and topology optimisation combined with shape optimisation is 30 and 60 degrees. The specimen optimised for topology and form has the shortest range in all directions, followed by the specimen optimised for topology alone. The original specimen’s range in all directions is greater than the ranges of the two optimised specimens mentioned above.

**Figure 16:**
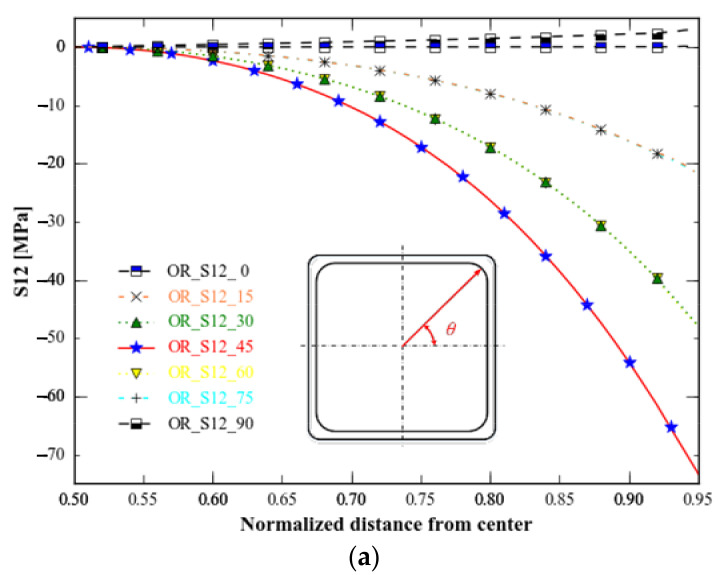

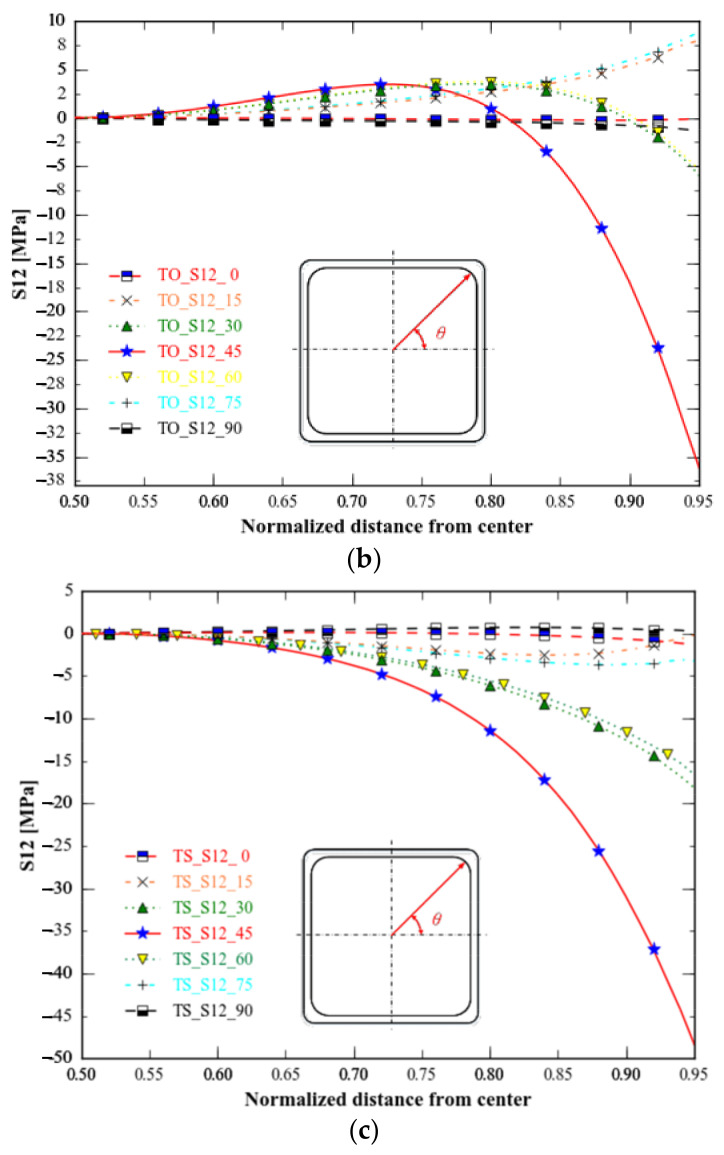
*S*_12_ of the three specimens in nine directions. (**a**) Type-A: unoptimised specimen; (**b**) type B: specimen with topology-only optimization; (**c**) type C: specimen with topology combined with shape optimisations.

The topology-optimised specimen has the lowest stress range for *S*_12_, followed by the topology and shape-optimised specimen (Figure 16). Stress distributions in all directions for topology-optimised specimen *S*_12_ and topology optimisation combined with shape optimisation differ from those observed for the original specimen, particularly those in the 45 degree direction: *S*_12_ from the centre to the boundary, where the original specimen and the specimen of topology optimisation combined with shape optimisation decrease, whereas the specimen of topology optimisation increases first and then decreases, which makes the stress distribution of the specimen optimised for topology and shape similar to that of the original specimen in different directions, but the former exhibits more minor stress change than the latter.

We calculated the average and variation of stresses in three distinct directions on three types of specimens to assess the stress homogeneity in all directions (Table 2). For *von Mises* stress, the uniformity of the specimen with topology optimisation and topology optimisation combined with shape optimisation is significantly better than that of the original specimen in the 30 and 45 degree directions, whereas, for *S*_11_, *S*_22,_ and *S*_12_, the uniformity of the specimen with topology optimisation and topology optimisation combined with shape optimisation is significantly lower in all directions than that of the original specimen.

### 3.4. Stress with Load Ratios

For the same observation area and load ratio, the *von Mises*, *S*_11_, *S*_22_, and *S*_12_ variances of type B and type C are significantly smaller than those of type A (Table 3). Furthermore, the stress uniformity of the type C (further shape-optimised) specimen decreases compared to that of the type B (topology-only) specimen.

## 4. Discussion

We found that topology and shape optimisations could improve the stress uniformity of the original specimen in the gauge zone. In the quantified section of the gauge area, the optimised specimen geometry produces a more uniform stress distribution, whose value is determined indirectly by simulations each time. Type C has the lowest standard deviation in *von Mises* stress for equal biaxial tensile stress, while type B has the lowest standard deviation in *S*_11_, *S*_22_, and *S*_12_. Type B and type C have markedly smaller *von Mises*, *S*_11_, *S*_22_, and *S*_12_ standard deviations than type A for the four load ratios. The above results show significantly improved stress uniformity of type B and type C in the gauge zone. A slight difference in the distribution of *von Mises, S*_11_, *S*_22_, and *S*_12_ could be attributed to the differences in the forms of type B and type C boundaries. With type C and type B both having the advantages of topology optimisation, the uniformity of *von Mises, S*_11_, *S*_22_, and *S*_12_ distributions are significantly improved compared to type A. In addition, the optimised specimens under biaxial tensile loading are suitable for different load ratios.

Shape optimisation of the specimen reduces the stress concentration at the intersection of the cross-arms, which is beneficial for preventing pre-failure outside of the gauge zone. Stress uniformity in the gauge zone is affected by the concentration of stresses at the cross-arm intersection [2,3,4,5,6,11]. Type C is slightly less uniform than type B in terms of the *S*_11_, *S*_22_, and *S*_12_ distributions because the changes in boundary shape hinder the load transfer improved by topology optimisation. However, to avoid pre-failure at the position of stress concentration, the boundary shape of the cross-arm intersection should be enhanced by employing shape optimisation. While we only optimised centre-reduced specimens, the shape optimisation method can also be applied to other forms of cruciform specimens, for example, inside concave fillets at the cross-arm intersection and slots on the cross-arms.

The stress distribution of the cruciform specimen in the gauge zone primarily rests on its structure [12,14,26]. According to optimization results from S. Demmerle, stress uniformity in the gauge area is markedly affected by the geometric structure of the cruciform specimen [8]. The specimen’s structure determines its force transmission characteristics, which remain stable with minor strains. Materials’ constitutive model and test objectives can be changed to optimised cruciform specimens that could adapt to different working conditions and research purposes. Specific materials are solely correlated with measuring strain rather than affecting the stress distribution for the same structure. Therefore, although we only discussed the stress uniformity in the gauge zone within the linear elastic material, we could apply the method to design cruciform specimens with various materials for minor strain, or even large strain, measurement by changing optimisation objectives through specific test requirements.

As a result of extending the fillet radius and optimizing the transition form, Gozzi reduces the stress concentration at the intersection of the cross-arm, but the stress distribution along the boundary of the cruciform specimen remains uneven [7]. Increasing the radius of the fillet could not solve the problem of stress concentration at the intersection of the cross-arms. In this research, shape optimization was accomplished by using the fillet radius as the starting point for constructing a new boundary that distributes stress at the lowest level possible. Further, slotting the cross arm changes the material distribution to enhance stress distribution in the gauge area, which is consistent with topology optimization [9]. After topology and shape optimization, the cruciform specimen’s gauge zone stress uniformity considerably improved, supporting the hypothesis that at least one material distribution (the optimised specimen in this study) can improve gauge zone stress uniformity in addition to slotting.

Unlike parameter optimisations [8,9,10,11,15,16,17,18], topology and shape optimisations improve stress uniformity by enhancing load transmission. Both of the above non-parametric optimisation methods are not bound by geometric parameters, increasing the chance of an optimal solution. As a result, non-parametric optimisation may be more effective than parametric optimisation for optimising cruciform specimens. Although we only carried out the numerical verification of the optimised specimen, the verified results demonstrate that the method improves the stress uniformity in the gauge zone, and reduces the stress concentration at the cross-arms intersection.

Using topology and shape optimisations, we designed a new cruciform specimen with significantly improved stress uniformity in the gauge zone. This method helps obtain reliable in-plan biaxial test results, by optimising the stress uniformity in the gauge zone, reducing the pre-failure caused by stress concentration.

## 5. Conclusions

We developed a novel centre-reduced cruciform specimen by optimising the topology and shape of the specimen using the finite element method. The results demonstrate a marked increase in stress uniformity compared to the original cruciform specimen. Additionally, the stress concentration at the intersection of the cross-arms is diminished. By improving the load path from the clamping end to the gauge zone, we improved the stress uniformity of the gauge zone, which may ensure the quality of the in-plane biaxial test results, and enable extensive strain tests.

Further research should be conducted to develop non-parametric optimisation methods coupled with specific working conditions to improve the stress uniformity of the gauge zone, and reduce the stress concentration at the cross-arm interface. As it stands, various optimisation techniques should be incorporated into the design of the cruciform specimen to address functional design issues, such as how to eliminate pre-failures at cross-arm intersections and determine nominal stress related to the applied load.

## Figures and Tables

**Table 1:** The average and variance of stress in different regions.

Stress	a/A	Type A		Type B		Type C	
		Average/MPa	Std/MPa	Average/MPa	Std/MPa	Average/MPa	Std/MPa
*von Mises*	0.1	396.39	0.01	417.63	0.00	390.72	0.00
	0.2	396.40	0.02	417.64	0.01	390.72	0.00
	0.3	396.44	0.07	417.66	0.06	390.73	0.01
	0.4	396.52	0.21	417.71	0.18	390.74	0.04
	0.5	396.73	0.56	417.82	0.44	390.77	0.11
	0.6	397.15	1.26	418.00	0.92	390.85	0.26
	0.7	397.81	2.41	418.27	1.63	390.96	0.49
	0.8	398.89	4.20	418.64	2.77	391.18	1.04
	0.9	400.36	6.96	418.99	4.73	391.23	1.88
	1.0	406.55	19.71	424.90	20.58	396.82	18.18
*S*_11_ *or S*_22_	0.1	396.40	0.11	417.63	0.07	390.69	0.04
	0.2	396.39	0.55	417.63	0.29	390.69	0.20
	0.3	396.41	1.37	417.66	0.66	390.68	0.49
	0.4	396.45	2.24	417.70	1.00	390.70	0.79
	0.5	396.51	3.79	417.80	1.50	390.72	1.36
	0.6	396.66	5.89	417.97	2.04	390.75	2.17
	0.7	396.91	7.87	418.22	2.70	390.79	3.02
	0.8	397.16	11.12	418.58	3.78	390.82	4.61
	0.9	397.06	14.87	418.61	5.78	390.35	6.79
	1.0	400.87	25.18	423.88	23.10	394.67	22.26
*S*_12_	0.1	0.00	0.18	−0.02	0.12	0.08	0.06
	0.2	−0.01	0.91	0.00	0.45	0.07	0.28
	0.3	0.01	2.15	0.00	0.99	0.07	0.66
	0.4	0.00	3.74	0.01	1.48	0.07	1.16
	0.5	0.03	6.23	0.01	1.98	0.07	2.00
	0.6	0.03	9.61	0.01	2.23	0.06	3.26
	0.7	0.02	13.43	0.00	2.45	0.04	4.71
	0.8	−0.01	18.59	−0.03	3.83	0.10	7.66
	0.9	0.01	25.50	0.02	7.84	0.22	11.73
	1.0	0.09	33.10	−0.01	12.50	0.10	15.83

**Table 2:** Stress distribution in different directions in the gauge area.

Stress	Direction	Type A		Type B		Type C	
		Average/MPa	Std/MPa	Average/MPa	Std/MPa	Average/MPa	Std/MPa
*von Mises*	0°	395.90	1.04	416.10	2.37	390.24	0.98
	15°	396.50	0.47	416.68	1.67	390.35	0.96
	30°	398.90	3.37	418.70	1.27	391.03	0.38
	45°	402.80	8.87	421.53	5.10	392.19	2.08
	60°	399.02	3.52	418.72	1.27	391.10	0.58
	75°	396.60	0.34	416.69	1.63	390.49	0.66
	90°	395.97	0.83	416.13	2.31	390.22	1.12
*S*_11_	0°	406.04	8.61	416.10	2.37	392.71	1.81
	15°	405.66	8.36	416.68	1.67	393.43	2.34
	30°	404.29	7.51	418.70	1.27	394.68	4.66
	45°	399.40	3.75	421.53	5.10	391.10	0.76
	60°	389.87	6.22	417.42	2.84	385.89	5.99
	75°	385.21	11.38	418.52	1.88	386.56	4.44
	90°	383.91	12.85	419.48	1.21	387.52	2.77
*S*_22_	0°	383.91	12.85	419.48	1.21	387.52	2.77
	15°	385.21	11.38	418.52	1.88	386.56	4.44
	30°	389.87	6.22	417.42	2.84	385.89	5.99
	45°	399.40	3.75	421.53	5.10	391.10	0.76
	60°	404.29	7.51	418.70	1.27	394.68	4.66
	75°	405.66	8.36	416.68	1.67	393.43	2.34
	90°	406.04	8.61	416.10	2.37	392.71	1.81
*S*_12_	0°	0.01	0.03	−0.12	0.09	−0.28	0.34
	15°	−6.51	6.45	2.28	2.19	−1.23	0.90
	30°	−14.11	14.06	1.24	2.10	−5.09	5.12
	45°	−21.68	21.73	−3.36	9.94	−11.20	13.34
	60°	−14.13	14.10	1.45	2.04	−4.65	4.72
	75°	−6.52	6.46	2.54	2.40	−1.83	1.36
	90°	0.01	1.40	0.13	0.68	0.28	0.33

**Table 3:** Stress uniformity in the gage zone under different load ratios.

a/A	Stress	Ratio	Average/MPa			Std/MPa		
			Type A	Type B	Type C	Type A	Type B	Type C
0.9	*von Mises*	1:4	451.00	445.10	416.00	28.43	17.36	16.76
		1:2	390.60	395.40	369.50	21.76	11.33	11.67
		3:4	371.90	385.70	360.20	13.02	6.54	6.21
		1:1	400.30	418.90	391.20	6.96	4.73	1.88
	S_11_	1:4	31.60	54.20	49.60	15.50	14.21	10.09
		1:2	153.40	175.60	163.20	9.44	9.44	6.06
		3:4	275.20	297.10	276.80	9.10	5.74	4.27
		1:1	397.00	418.60	390.30	14.87	5.78	6.79
	S_22_	1:4	464.70	469.00	438.10	28.72	16.66	15.79
		1:2	442.10	452.20	422.20	23.59	12.24	12.45
		3:4	419.60	435.40	406.30	18.85	8.27	9.34
		1:1	397.00	418.60	390.30	14.87	5.78	6.79
	S_12_	1:4	0.02	−0.15	0.28	16.73	11.65	12.35
		1:2	0.02	−0.10	0.25	19.43	9.18	11.53
		3:4	0.01	−0.04	0.22	22.38	7.71	11.73
		1:1	0.01	0.02	0.22	25.50	7.84	11.73
0.6	*von Mises*	1:4	468.13	451.97	421.74	13.11	5.23	5.80
		1:2	398.40	398.43	372.11	9.82	2.94	4.21
		3:4	371.83	385.86	360.70	5.50	1.32	2.26
		1:1	397.15	418.00	390.85	1.26	0.92	0.26
	S_11_	1:4	18.80	48.34	45.80	7.75	6.70	3.79
		1:2	144.75	171.55	160.78	4.67	4.98	2.15
		3:4	270.71	294.76	275.77	3.65	3.35	1.19
		1:1	396.66	417.97	390.75	5.89	2.04	2.17
	S_22_	1:4	477.08	474.13	442.72	12.77	5.19	5.88
		1:2	450.28	455.41	425.42	10.21	3.22	4.56
		3:4	423.48	436.69	408.12	7.84	1.67	3.29
		1:1	396.66	417.97	390.75	5.89	2.04	2.17
	S_12_	1:4	0.03	−0.18	0.19	6.11	3.06	3.31
		1:2	0.03	−0.12	0.13	7.24	2.47	3.15
		3:4	0.03	−0.06	0.06	8.41	2.15	3.26
		1:1	0.03	0.01	0.06	9.61	2.23	3.26
0.3	*von Mises*	1:4	480.01	457.61	426.15	3.50	1.41	1.49
		1:2	404.39	401.13	374.30	2.56	0.71	1.06
		3:4	372.98	386.33	361.19	1.40	0.18	0.56
		1:1	396.44	417.66	390.73	0.07	0.06	0.01
	S_11_	1:4	10.41	44.07	42.61	2.12	1.91	1.04
		1:2	139.08	168.60	158.64	1.26	1.47	0.60
		3:4	267.74	293.13	274.67	0.84	1.04	0.29
		1:1	396.41	417.66	390.68	1.37	0.66	0.49
	S_22_	1:4	485.13	478.04	445.86	3.32	1.24	1.49
		1:2	455.56	457.91	427.49	2.60	0.71	1.13
		3:4	425.99	437.78	409.13	1.93	0.36	0.79
		1:1	396.41	417.66	390.68	1.37	0.66	0.49
	S_12_	1:4	0.02	−0.18	0.22	1.35	0.66	0.50
		1:2	0.02	−0.12	0.14	1.61	0.76	0.57
		3:4	0.01	−0.06	0.07	1.88	0.87	0.66
		1:1	0.01	0.00	0.07	2.15	0.99	0.66
